# Crystal clear or tin ear: how do medical students interpret derogatory comments about patients and other professionals?

**DOI:** 10.3402/meo.v21.31221

**Published:** 2016-07-13

**Authors:** Sara G. Tariq, Carol R. Thrush, Molly Gathright, John J. Spollen, James Graham, Jeannette M. Shorey

**Affiliations:** 1 Department of Internal Medicine, University of Arkansas for Medical Sciences, Little Rock, AR, USA; 2 Department of Surgery, University of Arkansas for Medical Sciences, Little Rock, AR, USA; 3 Department of Psychiatry, University of Arkansas for Medical Sciences, Little Rock, AR, USA; 4 Division of Academic Affairs, University of Arkansas for Medical Sciences, Little Rock, AR, USA

**Keywords:** learning environment, clerkships, professionalism

## Abstract

**Purpose:**

To assess the learning environment at our medical school, third-year medical students complete an 11-item survey called the Learning Environment for Professionalism (LEP) at the end of each clerkship. The LEP survey asks about the frequency of faculty and resident professional and unprofessional behaviors that students observed; two of the items specifically address derogatory comments. This study used focus group methodology to explore how medical students interpret the derogatory comments they reported on the LEP survey.

**Methods:**

Seven focus groups were conducted with 82 medical students after they completed the LEP survey. Analysis of focus group transcripts was performed to better understand the nature and meaning that students ascribe to derogatory comments.

**Results:**

The study results provide insights into the types of derogatory comments that medical students heard during their clerkship rotations, why the comments were made and how they were interpreted. Emergent themes, labeled by the authors as 1) ‘onstage-offstage’, 2) ‘one bad apple’, and 3) ‘pressure cooker environment’, highlight the contextual aspects and understandings ascribed by students to the derogatory comments. Incidentally, students felt that the comments were not associated with fatigue, but were associated with cumulative stress and burn-out.

**Conclusions:**

The results suggest students have a clear understanding of the nature of unprofessional comments made by role models during clerkships and point to important systems-related issues that could be leveraged to improve clinical learning environments.

Professionalism is expected of physicians by their patients, their students, and the entire health care team. National and international physician organizations and medical school accreditors have codified the importance of consistently professional behavior on the part of physicians. Medical schools have responded with various strategies to foster and measure the professional conduct of their learners and faculty.

One strategy to understand, analyze, and foster professionalism that we have implemented at the University of Arkansas for Medical Sciences is to ask our third-year medical students to complete a survey (Learning Environment for Professionalism (LEP) survey) (1) about their observations of professionalism-related behaviors in the learning environment during the required clerkships. The LEP survey data are analyzed and presented annually to all relevant education leaders at our institution (e.g., clerkship directors, residency program directors, and department chairs). The results have consistently shown that the most common unprofessional behaviors noted by our students are related to physicians making derogatory comments about other medical professionals and derogatory comments about patients. In the year prior to the study, the percentage of students that reported never observing such derogatory comments ranged across clerkships from 8 to 68% indicating such comments were unfortunately commonplace. Similar results have been found using a survey that contained almost identical items at a different medical school (2).

During the course of providing annual LEP survey summary reports to institutional leaders at our school, concerns were raised about what kinds of unprofessional behaviors the students actually saw during their clerkships and how students interpreted such behaviors. In addition, there was concern that because students were naive to the clinical learning environment, they may be misconstruing physician comments as ‘unprofessional’ when they were intended to be constructive criticism.

Previous reports have described how the use of derogatory and cynical humor directed towards patients is viewed as unprofessional by medical students (3), and by residents and attendings alike (4). Such comments may occur when physicians have not developed more positive coping strategies for managing their reactions and feelings about challenging or ‘difficult’ patients, such as those who are non-compliant or those who are perceived as self-abusers (excessive drinkers, drug users) (3). Derogatory humor, and the acceptance of and eventual use of it by medical students, may also serve as part of the professional acculturation process whereby students move from ‘outsider’ to ‘insider’ status (5, 6). Nonetheless, derogatory statements are disrespectful and dehumanizing and erode a sense of professionalism and civility in the clinical workplace (7).

Our goal in this study was to gain a deeper understanding of the setting, the meaning, the characters, and the circumstances that surrounded voicing of the derogatory comments students documented in the LEP survey. In addition, we wanted to know if the students thought that factors like fatigue might have contributed to the unprofessional behaviors.

## Methods

We used focus group methodology for this study because it is interactive and promotes discussion of sensitive topics. This format encourages students to interact and talk with each other, ask questions, share stories, and comment on their experiences and points of view (8). The study was formally approved as exempt by our institutional review board (IRB). Informed consent was verbally obtained and a waiver of signed informed consent was obtained so as to reduce any risk of identification of participants. An incentive to participate included free lunch for participants.

### Participants and procedures

Seven focus groups, one for each core clerkship, were held in December 2011, immediately after students completed the LEP survey at the end of the clerkship. Approximately 18–24 students per focus group (depending on rotation) were invited to participate in the discussion over the lunch hour. A total of 82 students participated including 12 in family medicine, 7 in geriatrics, 22 in internal medicine, 16 in obstetrics-gynecology, 4 in pediatrics, 6 in psychiatry, and 15 in surgery. All students from a particular rotation were in the same focus group.

Four faculty facilitators shared the work of conducting the seven focus groups. Each group had one facilitator and one note-taker. The focus groups were led by a facilitator who did not hold a position in the specific department affiliated with the clerkship and who had experience in leading focus group discussions. Focus group sessions were held in a classroom at the medical school or in a conference room in the hospital. Each session was recorded using a digital recorder. The students were informed that the session would be recorded and that the facilitator and the note-taker would be the only two people who would have access to the recording. Audio files were transcribed for analysis and subsequently destroyed.

The research team developed a common script and template for all facilitators to use in conducting the focus groups. The facilitator initiated the session with the following opening statement: ‘We are interested in gaining a better understanding about your thoughts when you were completing the LEP survey. We have been using this survey at UAMS for several years. We would like to gain insights into what you were recollecting and thinking about when you were completing the survey items’. Further, the facilitators explained there were three areas in which we wanted to focus discussion: 1) attendings/residents making derogatory comments about patients and families, 2) attendings/residents making derogatory comments about other providers, and 3) whether derogatory comments were associated with fatigue.

A final transcript of each focus group was produced by the respective focus group facilitator who reconciled the note-takers’ additional input with the audio transcripts. Subsequently, the research team convened a series of meetings during which each transcript was read aloud. First, each member of the team individually wrote down the themes he or she identified. These were shared aloud. Non-verbal behaviors of students observed by facilitators helped inform the focus group analysis discussions. Then, through reflective conversations, the team sought to understand and distill the prominent themes within and across clerkships. This process was repeated at a later time to ensure that the salient themes remained consistent.

## Results

Focus group discussions with the students indeed provided much insight into the learning environment. Overall, the vast majority of the physicians behaved quite professionally and were seen as positive role models. However, it was quite clear that negative comments were accurately understood as either derogatory or as constructive criticism. The students clearly distinguished the two and reported that non-verbal communication (e.g., eye rolling, tone of voice, body language) as well as jargon helped distinguish whether a comment was derogatory or not.

They spoke frequently about appreciating faculty members who thought aloud about clinical reasoning, especially in the setting of mistakes. These were important learning moments. However, the learning fell flat if the students were distracted by derogatory comments or editorial comments of a personal nature. The students regretted the distractions.

In addition, students described a graduated acceptability of derogatory remarks. They reported that derogatory remarks were never acceptable in front of patients but were often tolerated when made in front of students and were ‘OK’ to voice with peers. The derogatory comments were not necessarily associated with fatigue, as was initially suspected. Rather, the students identified ‘cumulative stress’ and ‘burn-out’ as sources of the negative comments.

Extensive analysis of the derogatory comments across all clerkships revealed several themes. These themes provide some insight into why the comments were made and how they were interpreted. The themes that emerged were coined 1) ‘onstage-offstage’, 2) ‘one bad apple’, and 3) ‘pressure cooker environment’.

### Onstage-offstage

In most cases when derogatory comments about patients were heard by students, they occurred outside the patient's room. The students typically forgave this behavior. They often noted that this was a form of venting about patients who were ‘non-compliant’. When physicians were ‘on-stage’, they behaved professionally, even with compassion. As long as the physicians were ‘off-stage’, students reported that it was more tolerable to make derogatory comments about patients as a way of venting their frustration with ‘difficult patients’.

I said ‘occasionally’ – not ‘never’ [on the survey] but not that frequent. The comments were more of a venting technique – said in a workroom or someplace like that – never directly to the patient. I'm not saying that that's OK – it's unprofessional – but my experiences of hearing derogatory remarks were after having worked with a particularly spiteful Mom – really mean-spirited. After a while you can only take so much verbal abuse from parent without having to vent a little bit so I feel like most of my experiences were venting.

Some students felt flattered when they were privy to the derogatory ‘off-stage’ remarks because they believed this made them more a part of the ‘club’. Other students considered this type of behavior as inappropriate, even if the patient was under anesthesia or out of ‘earshot’ when such comments were made.

Students also cited two common reasons for frustration leading to derogatory comments: patient's poor health-related choices and treatment non-adherence. Often, derogatory comments tended to be focused towards people with obesity, for example, ‘they did this to themselves’. Other comments, such as the following quote, had a more paternalistic tone, aiming frustration towards patients with lower income or lower literacy.

Mine (examples) were few and far between … usually regarding the intelligence of parents making certain decisions like a parent refusing vaccinations.

### One bad apple

Students often identified that there was only ‘one or two’ physicians on the clerkship rotation who exhibited unprofessional behavior; however, these few were enough to detrimentally affect the learning environment. Even if students observed one event, it affected their perception of the whole group. In a few clerkships, students recognized a general disregard for the team, noting that relationships between all levels of hierarchy are challenged. For example, students were criticized for being ‘stupid’. One was told insultingly that he would ‘make a great pediatrician someday’. Even though these comments were fairly isolated, they tainted the students’ perception of that clerkship.

Some of the attendings are nice. The bad ones are a small percentage, but they make it hard for everyone.

This ‘bad apple’ phenomenon is echoed by a study published in the organizational behavior literature. Teams that had one member who bullied others were much more likely to experience conflict, to experience poor communication, and to have individuals refusing to cooperate with one another. In addition, it was found that negative behavior outweighed positive behavior. One bad apple could spoil the barrel, but one or two professional team members could not ‘unspoil’ it. In less hierarchical teams where members are more equal, the other team members were more likely to address the negative person (9). In organizations like academic medical centers, it is unlikely that students who are at the bottom of the academic hierarchy will be empowered to address the issue with the negative member.

### Pressure cooker environment

A pressure cooker environment is defined as one that is fraught with emotional or social pressures. It is extremely demanding, and workers in this environment are consistently under stress with risk of burn-out. Certainly, the inpatient service of an academic medical center, in particular, meets this criterion. Students in all clerkships but one recognized this pressure. The consequence of this pressure was demonstrated in several ways, but was most distinct in interactions where consultation among specialties was needed and in the language, behaviors, and attitudes manifested around communicating or ordering a consult from another department. Students noted these interactions were sometimes disrespectful. For example, decisions made by the Emergency Medicine physicians were mocked as ‘these guys don't know what they're doing’. All doctors in other specialties are ‘stupid’. Radiology was referred to as the ‘shadow science’. Students expressed that it was easier to judge doctors outside the service – this judgment was revealed more often when something went wrong on a consult.

Everybody has their service (they talk badly about) … it usually comes out when something goes wrong with a patient that you are responsible for, and you have consulted a service and they don't do something right.

## Discussion

Based on in-depth conversation that took place in the focus groups, students indeed understood the nuances and meaning surrounding the derogatory comments made about other providers and patients. In addition, most didn't ascribe the negative comments as being due to physician fatigue but rather due to other factors such as cumulative stress and burn-out.

Much has been written in the literature about the hidden curriculum and the erosion of empathy that occurs in the clinical years. This literature suggests that the clerkship year represents a turning point, where students begin to internalize unprofessional behaviors and consider them as acceptable. Conversations from our students revealed that they had not yet fully internalized the acceptability of making derogatory comments about patients or other healthcare providers. These students were at an important turning point in the development of their professional identity. They clearly separated themselves from the negative behaviors, yet they appreciated being included, flattered even in some cases, when they were allowed to ‘listen in’ on the venting occurring away from patients. This point in the students’ identity development may be the best time for clerkship directors to implement some format of reflective learning. Reflective learning, via facilitated conversations and writing exercises, has been shown to improve professionalism and can contribute to continuous practice improvement (10). Several schools have initiated a model where students submit (professional and unprofessional) stories on-line from their experiences in the clerkships and then reflect on them with a faculty member in a confidential and non-threatening environment (11–14).

Some schools have taken a proactive approach to address unprofessional behaviors manifest by specific faculty. Our institution has adopted such a system, modeled after one developed at Vanderbilt University School of Medicine (15). In this system, any member of the healthcare team may log a faculty member's unprofessional behavior in a web-based reporting system. A council of faculty peers assesses the severity and pattern of the reports about individual physicians and takes action if the reports reflect a pattern of repeatedly unprofessional behavior or a single egregious act.

It is important that faculty and residents understand that ‘on-stage’ means they are being watched and emulated by students. Thus, conversations in which frustrations are vented should likely take place without students present. If they do occur in front of students, they should be done in a respectful manner. Administrators and educational leaders may want to invest time in faculty development that addresses such communication issues (3). If these behaviors manifest more with accumulated stress and burn-out, as our focus groups found, then departments might also benefit from investing in faculty and resident wellness programs.

## Conclusion

Professionalism is learned most effectively through the influence of clinicians that students encounter during their education (16). This focus group study showed that students can indeed recognize a derogatory comment. They understand the nuances and the subtleties in the communication that might make a comment derogatory. In addition, the cross-clerkship themes that emerged from this study point to concerns that can be addressed via curricular innovation and programmatic change to improve the learning environment.
